# Hypoxia-Targeting Strategies in Radiotherapy and Nitroimidazole-Based Radiosensitizers: A Narrative Review and Translational Perspectives

**DOI:** 10.3390/cimb48090863

**Published:** 2026-08-25

**Authors:** Enrico Rosa, Bruno Fionda, Maria Vaccaro, Alessio Giuseppe Morganti, Francesco Marampon, Stefano Arcangeli, Monica Mangoni, Marco De Spirito, Maria Antonietta Gambacorta, Luca Tagliaferri

**Affiliations:** 1 Dipartimento di Neuroscienze, Sezione di Fisica, Università Cattolica del Sacro Cuore, 00168 Rome, Italy; 2 Dipartimento di Diagnostica per Immagini e Radioterapia Oncologica, UOC Fisica per le Scienze della Vita, Fondazione Policlinico Universitario A. Gemelli IRCCS, 00168 Rome, Italy; 3 Dipartimento di Diagnostica per Immagini e Radioterapia Oncologica, UOC Degenze di Radioterapia Oncologica, Fondazione Policlinico Universitario A. Gemelli IRCCS, 00168 Rome, Italy; 4 Radiation Oncology, IRCCS Azienda Ospedaliero-Universitaria di Bologna, 40126 Bologna, Italy; 5 Department of Radiological Sciences, Oncology, and Anatomical Pathology, Sapienza University of Rome, Policlinico Umberto I, Viale del Policlinico 155, 00161 Rome, Italy; 6 Radiation Oncology Unit, Fondazione IRCCS San Gerardo dei Tintori, 20900 Monza, Italy; 7 Biostatistics and Clinical Trial Center, Fondazione IRCCS Policlinico San Matteo, 27100 Pavia, Italy; 8 Radiotherapy Unit, Department of Experimental & Clinical Biomedical Sciences, University of Florence, 50134 Florence, Italy; 9 Dipartimento di Diagnostica per Immagini e Radioterapia Oncologica, UOC Servizio di Radioterapia Oncologica, Fondazione Policlinico Universitario A. Gemelli IRCCS, 00168 Rome, Italy; 10 Dipartimento di Scienze Radiologiche ed Ematologiche, Università Cattolica del Sacro Cuore, 00168 Rome, Italy

**Keywords:** radiotherapy, tumor hypoxia, radiosensitizers, nitroimidazoles

## Abstract

**Background:** Tumor hypoxia is a major determinant of radioresistance, limiting oxygen-mediated fixation of radiation-induced DNA damage and promoting metabolic adaptation, tumor aggressiveness, and treatment failure. Nitroimidazole derivatives and related hypoxia-targeting compounds have been investigated as radiosensitizers because of their oxygen-mimetic properties and selective activation under low-oxygen conditions. **Methods:** A structured narrative review was conducted to evaluate recent evidence on hypoxia-targeting strategies and nitroimidazole-based radiosensitizers combined with radiotherapy. PubMed and Scopus were searched in March 2026 using terms related to radiotherapy and nitroimidazoles. Studies published within the last five years were included if they investigated hypoxia-targeting compounds, radiosensitization strategies, or dose–response relationships relevant to radiotherapy. Preclinical, computational, and clinical studies were considered. **Results:** Sixteen studies were included, comprising preclinical, computational, and clinical investigations. Most studies were preclinical and evaluated in vitro cell lines, murine tumor models, or xenografts across multiple tumor types, including head and neck cancer, glioblastoma, breast cancer, colorectal cancer, cervical cancer, renal carcinoma, pancreatic cancer, and ovarian-related models. Nitroimidazole-based and related hypoxia-targeting strategies generally enhanced radiation response under hypoxic conditions, with sensitizer enhancement ratio values ranging from approximately 1.09 to 1.65. In vivo studies reported reductions in tumor growth or tumor volume when radiosensitizers were combined with radiotherapy. Clinical evidence, mainly in head and neck squamous cell carcinoma, showed variable effects on locoregional outcomes. **Conclusions:** Current evidence supports the biological relevance of hypoxia-targeting strategies for improving radiotherapy response. Nitroimidazole-based compounds show consistent radiosensitizing effects in preclinical models, but clinical translation remains heterogeneous. Future studies should integrate hypoxia imaging, standardized endpoints, advanced drug delivery systems, and clinically relevant radiotherapy models to better define their role in modern radiation oncology.

## 1. Introduction

Radiotherapy (RT) is widely used in oncology and represents a cornerstone treatment across multiple tumor types and clinical settings. The biological effectiveness of RT is mainly driven by DNA damage induced by ionizing radiation and by the generation of reactive oxygen species (ROS), which ultimately lead to tumor cell death. However, RT effects are not limited to DNA damage. Radiation also induces significant alterations in cellular metabolism and survival pathways, affecting mitochondrial function, energy production, and redox balance. These changes contribute to both tumor control and treatment resistance [1]. Radiation-induced metabolic alterations can reprogram tumor cells, increasing their dependence on specific energy pathways and modulating autophagy and stress response mechanisms. These non-DNA effects play an important role in long-term outcomes and may explain part of the variability in RT response.

However, the presence of hypoxic regions within tumors remains one of the main limitations to RT efficacy. Tumor hypoxia is caused by abnormal vascularization and rapid tumor growth, which reduce oxygen availability within the tissue. Reduced oxygen levels impair the fixation of radiation-induced DNA damage, thereby promoting radioresistance. Hypoxia can also enhance radiation-induced metabolic adaptations, further shifting tumor cells toward glycolysis and survival pathways. This represents a relevant biological constraint that influences treatment response across multiple tumor types and disease stages. Hypoxia not only limits RT-induced cytotoxicity but is also associated with increased tumor aggressiveness, metabolic reprogramming, and altered signaling pathways. It also promotes resistance to other treatments, including chemotherapy and immunotherapy. Importantly, this phenomenon is observed across different RT modalities, including external beam RT, brachytherapy, and radionuclide-based approaches, indicating that hypoxia represents a transversal challenge in radiation oncology that requires targeted and modality-independent solutions [2].

Although most of the available evidence reviewed in this work concerns external beam radiotherapy, hypoxia may also be relevant in localized treatments such as interventional radiotherapy (modern brachytherapy). In this setting, very high doses are delivered close to the tumor with steep dose gradients, so hypoxic areas located in lower-dose regions may respond differently from better-oxygenated or higher-dose regions. This spatial heterogeneity may influence treatment response and local control. Although extracellular-vesicle-mediated signaling has been proposed as a possible contributor to biological responses across heterogeneous dose distributions, its role in hypoxia-related radioresistance remains insufficiently defined and was not directly addressed by the studies included in this review [3,4,5].

**Figure 1:**
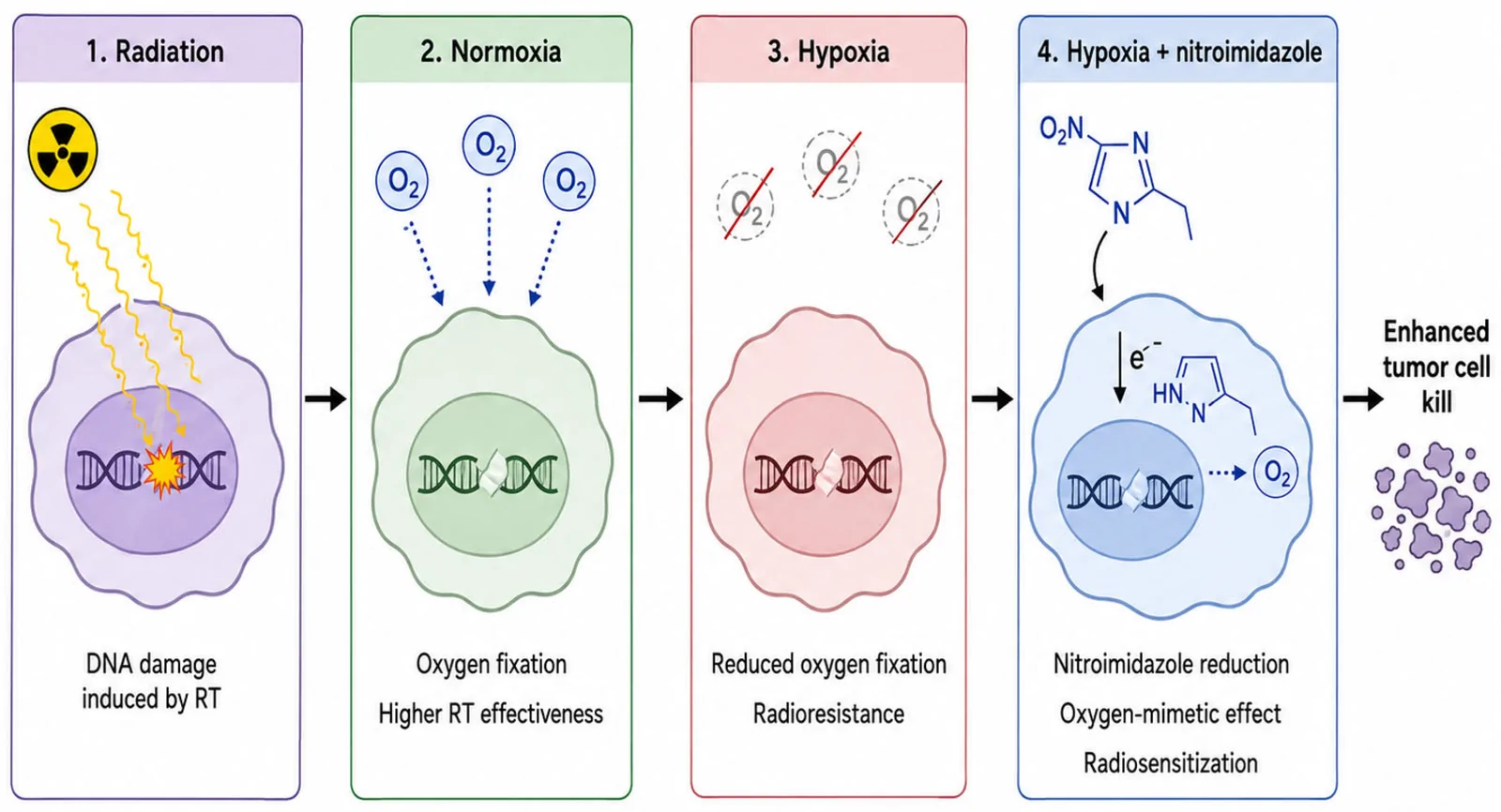
Simplified schematic representation of hypoxia-related radioresistance and nitroimidazole-based radiosensitization.

In addition, the identification of compounds capable of radiosensitizing hypoxic tumor regions has become a central objective in radiation oncology. Among these, nitroimidazole derivatives represent one of the most extensively investigated classes of hypoxia-selective radiosensitizers. Nitroimidazoles are a family of heterocyclic compounds characterized by the presence of a nitro group (-NO_2_) bound to an imidazole ring, which confers electron-affinic properties and enables selective bioreduction in low-oxygen conditions. Under hypoxic conditions, these molecules undergo enzymatic reduction to form reactive intermediates that can bind to cellular macromolecules, including DNA, thereby stabilizing radiation-induced damage and enhancing cytotoxic effects. This oxygen-mimetic behavior allows nitroimidazoles to compensate for the lack of molecular oxygen during RT, effectively increasing the fixation of DNA lesions and improving treatment efficacy. However, variability in radiosensitizing efficacy across different compounds, experimental models, and oxygenation conditions remains an important limitation, highlighting the need for comparative analyses [6,7,8]. A simplified schematic representation of hypoxia-related radioresistance and nitroimidazole-based radiosensitization is shown in Figure 1.

The studies analyzed in this work confirm that these compounds can significantly increase the sensitizer enhancement ratio (SER), modulate tumor growth kinetics, and improve overall treatment response across multiple experimental settings. Moreover, their selective activation in hypoxic conditions represents a key advantage, as it allows for enhanced tumor targeting while potentially sparing well-oxygenated normal tissues, thereby improving the therapeutic ratio of RT [9]. In addition, nanocarriers and hybrid systems can enhance tumor uptake, prolong retention time, and provide controlled release of the active compound, further increasing treatment efficacy. These advances are particularly relevant in heterogeneous tumors, where spatial variations in oxygenation and microenvironmental conditions require highly adaptable and targeted therapeutic strategies [10,11].

The effectiveness of hypoxia-targeting radiosensitizers depends not only on the presence of hypoxic tumor regions, but also on factors that influence drug delivery and biological activity. These include the degree and spatial distribution of hypoxia, tumor perfusion, compound uptake, retention time, and the ability of the radiosensitizer to reach poorly oxygenated tumor areas. In addition, differences in experimental models and irradiation conditions may affect the observed radiosensitizing effect and limit comparison across studies. These aspects are particularly relevant when translating preclinical findings into clinical practice and highlight the need for standardized experimental conditions and quantitatively comparable endpoints [12].

The present review aims to provide a comprehensive evaluation of recent developments in hypoxia-targeting radiosensitizers, with particular emphasis on nitroimidazole-based compounds, their mechanisms of action, and their potential for translation from preclinical research to clinical RT applications. In addition, modern radiotherapy techniques differ substantially from those used in earlier studies. Restricting the analysis to the recent literature allows evaluation of radiosensitization strategies within current clinical practice. While the use of nitroimidazoles is well established and supported by earlier studies, including those from the 1990s, this review focuses on their integration with contemporary RT techniques and delivery systems.

## 2. Materials and Methods

A literature search was conducted in March 2026 using the PubMed and Scopus databases. The search strategy combined terms related to radiotherapy and nitroimidazole-based hypoxia targeting, using the following search string: “radiotherapy” AND “nitroimidazoles”. Studies published within the previous five years were considered in order to focus on recent developments in radiotherapy and drug delivery technologies. After removal of duplicate records, titles and abstracts were screened for relevance to radiotherapy, tumor hypoxia, and hypoxia-targeting radiosensitization. Potentially eligible studies were then assessed by full-text review. Experimental, computational, and clinical studies were included if they evaluated nitroimidazole-based radiosensitizers or broader hypoxia-targeting strategies in combination with radiotherapy, or if they contributed quantitative or mechanistic information on dose–response relationships and treatment effectiveness. Studies were excluded if they were not related to radiotherapy or tumor hypoxia, did not evaluate a relevant hypoxia-targeting or radiosensitizing approach, were review articles, were published outside the predefined five-year period, or had no available full text. Following full-text assessment, 16 studies were included in the qualitative synthesis.

These were categorized according to study type (preclinical, computational, or clinical). Therapeutic endpoints included sensitizer enhancement ratio (SER), tumor growth or volume, survival-related parameters, and modeling-based estimates of treatment response.

Data extraction included study design, experimental model (in vitro, in vivo, or clinical), tumor type, type of radiosensitizer or hypoxia-targeting agent, and quantitative therapeutic outcomes. When multiple experimental conditions were reported, these were analyzed separately to preserve variability. Three tables were generated: two summarizing the characteristics of the included preclinical and clinical studies, respectively, and one reporting quantitative outcomes. Numerical data explicitly reported in the original studies were included in the quantitative table. When numerical values were not reported in the text or tables but could be reliably inferred from graphical data, they were estimated by visual interpolation of the plotted values and axis scales. These estimates were included only when the graphical representation allowed a sufficiently clear numerical approximation and were specifically identified as estimated values in the quantitative synthesis. Qualitative findings contributed to the descriptive synthesis.

Given the heterogeneity of models and endpoints, a semi-quantitative comparative analysis was performed when comparable endpoints were available, particularly for preclinical studies reporting SER. Clinical studies reporting survival-related outcomes (e.g., hazard ratios, HR) were analyzed separately.

Due to variability across studies, formal pooled meta-analysis was not feasible. Therefore, a qualitative and semi-quantitative synthesis was conducted to identify consistent trends in radiosensitization, tumor control, and the role of nitroimidazole-based compounds in overcoming hypoxia-induced RT resistance, with particular attention to their therapeutic impact.

The present work was conducted as a structured narrative review based on a systematic literature search. Due to the heterogeneity of study designs, endpoints, and experimental models, a formal systematic review or meta-analysis was not performed.

## 3. Results

A total of 16 studies were included in the qualitative synthesis after screening and eligibility assessment, comprising preclinical, computational, and clinical investigations. The characteristics of the included studies are summarized in Table 1; in Table 2 we included specific clinical studies while quantitative therapeutic outcomes extracted from selected studies are reported in Table 3.

**Figure 2:**
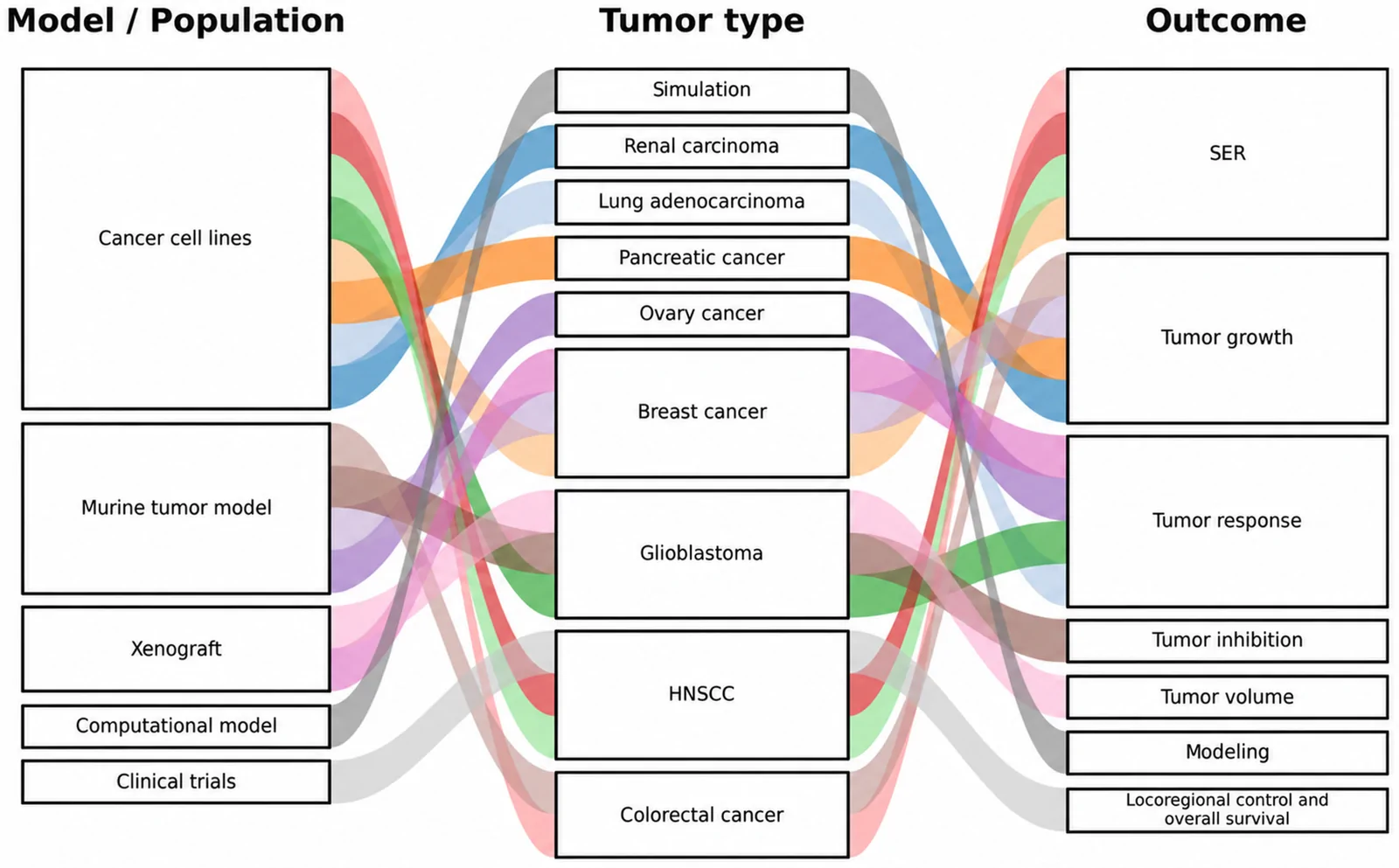
Alluvial plot illustrating the distribution of included studies according to model, tumor and outcome categories.

The included studies were categorized according to study type. Most studies were preclinical (n = 12), including both in vitro and in vivo models. A smaller number of studies were clinical (n = 3), while one study was based on a computational modeling approach. The distribution of studies across model/population, tumor type, and outcome categories is illustrated in Figure 2.

Preclinical studies included a range of experimental models. In vitro investigations were primarily based on established cancer cell lines, including FaDu, HCT116, MDA-MB-231, HeLa, and renal carcinoma cell lines [7,14,22,23]. In vivo studies were conducted using murine tumor models and xenografts, including CT26 colorectal cancer, 4T1 breast cancer, and glioblastoma models [18,19,23,24]. One study combined radionuclide-based approaches with murine models [15]. In addition, one study employed a computational framework to model dose–response relationships under hypoxic conditions [20]. The tumor types investigated included head and neck squamous cell carcinoma (HNSCC), glioblastoma, breast cancer, pancreatic cancer, colorectal cancer, cervical cancer, renal carcinoma, and ovarian-related models (Table 1).

Preclinical therapeutic studies evaluated radiosensitization using endpoints such as sensitizer enhancement ratio (SER), tumor growth, tumor volume, and tumor inhibition. SER was reported in multiple in vitro studies, with values ranging from 1.09 to 1.65 depending on the compound and experimental conditions [7,14,17]. Additional SER values were reported for gold cluster–based radiosensitizers, with values of 1.43 and 1.16 under hypoxic conditions, and 1.33 under normoxic conditions [22]. Tumor growth and tumor volume were the most frequently reported endpoints in in vivo models. Quantitative differences between RT alone and combined treatments were observed across studies; for example, tumor volume values of 882% for RT alone compared to 288% for combined treatment were reported in a glioblastoma xenograft model [24]. Tumor inhibition and growth delay effects were also reported in murine models [18,19,21,23].

Clinical studies evaluated treatment-related outcomes using time-to-event endpoints, particularly locoregional outcomes. In the NIMRAD randomized phase III trial, the addition of nimorazole to IMRT did not significantly improve freedom from locoregional progression in patients with hypoxia-high HNSCC (adjusted HR, 0.72; 95% CI, 0.36–1.44). Other clinical studies reported hazard ratios for locoregional failure of 0.51 (0.27–0.95) and 1.16 (0.84–1.59), depending on study design and treatment conditions [25,26,27] (Table 3).

Most studies were conducted using external beam radiotherapy. A limited number of studies evaluated alternative modalities, including radionuclide-based radiotherapy (Lu-177) [15] and simulated RT conditions in computational models [20] (Table 1).

Overall, therapeutic endpoints varied across studies in terms of type and measurement scale, including continuous variables (e.g., SER), relative tumor growth or volume changes, and survival-related outcomes (Table 3). The relationships between model/population, tumor type, and outcome categories are illustrated in Figure 2.

## 4. Discussion

### 4.1. Mechanistic Basis of Hypoxia-Targeting Radiosensitization

The present analysis further supports the role of tumor hypoxia as a limiting factor in radiotherapy efficacy, in line with the radiobiological framework outlined in the Introduction. Reduced oxygen availability in hypoxic tumor regions impairs the fixation of radiation-induced DNA damage, thereby decreasing the effectiveness of ionizing radiation. This effect, commonly described by the oxygen enhancement ratio (OER), represents a consistent constraint across different radiotherapy modalities, including external beam radiotherapy, brachytherapy, and radionuclide-based approaches [4]. Despite these findings, translation into clinical practice remains limited. The higher and more homogeneous radiosensitizing effects observed in simplified experimental models may not fully reflect the conditions encountered in vivo. In addition to spatial heterogeneity of tumor hypoxia, radiosensitizer activity may be influenced by several pharmacological and biological factors, including systemic drug exposure, the area under the concentration–time curve (AUC), extravascular transport into poorly perfused hypoxic regions, tumor uptake and retention, treatment schedule relative to irradiation, and the extent to which compound activity depends on local oxygenation. These factors may substantially affect the concentration and activity of the radiosensitizer within hypoxic tumor subregions and should therefore be considered when translating preclinical findings into clinical settings.

### 4.2. Preclinical Evidence

The results of the present analysis provide quantitative support for the radiosensitizing effect of hypoxia-targeting strategies across different experimental settings. In particular, the consistent increase in sensitizer enhancement ratio (SER), together with measurable reductions in tumor growth and tumor volume observed in preclinical models, confirms the biological activity of nitroimidazole-based compounds and other hypoxia-targeting strategies under hypoxic conditions [7,14,17]. These effects were reproduced across multiple tumor types and experimental systems, including in vitro cell lines, murine models, and xenografts, suggesting a coherent and reproducible therapeutic pattern despite the heterogeneity of study designs. In addition, the inclusion of computational modeling approaches further supports the mechanistic consistency of these findings under controlled conditions [20]. Notably, while OER values in mammalian cells typically range between 2–3, the SER values observed for nitroimidazoles are generally lower, reflecting their role as partial oxygen mimetics while still providing meaningful radiosensitization under hypoxic conditions. These findings are consistent with their mechanism of action and support their capacity to effectively enhance radiation response in oxygen-deprived tumor regions. In this context, the integration of hypoxia imaging (e.g., FMISO, FAZA PET) into treatment planning may enable improved patient selection and spatially optimized dose escalation strategies, further enhancing the therapeutic potential of hypoxia-targeting radiosensitizers [28].

### 4.3. Clinical Evidence and Translational Relevance

From a translational perspective, the availability of clinical data reporting hazard ratios for locoregional outcomes provides preliminary evidence that these effects may extend beyond preclinical models [26,27]. However, the limited number of clinical studies and the variability of reported endpoints still restrict the strength of these conclusions. Overall, the integration of preclinical, computational, and clinical evidence supports the potential of hypoxia-targeting radiosensitizers as a complementary strategy to improve radiotherapy response, particularly in hypoxic tumor subregions where conventional treatments are less effective.

However, the clinical evidence identified through this review remains very limited. Only three clinical studies were included, all conducted in patients with HNSCC, and their results were not fully consistent. This limitation should be interpreted in the context of the review methodology, including the restricted publication period and the relatively small number of studies included overall. Therefore, the present review does not allow firm conclusions to be drawn regarding the overall extent of clinical development of hypoxia-targeting radiosensitizers. Larger and more comprehensive clinical evidence syntheses are needed to better define their potential across different tumor types and treatment settings.

Despite these limitations, available clinical data suggest that hypoxia-targeting approaches may influence treatment-related outcomes in selected patients. Although the impact on overall survival appears more variable, the available evidence indicates that modulation of hypoxia may influence treatment response in selected patient populations. These observations are consistent with previous clinical investigations of nitroimidazole derivatives such as nimorazole, which have provided evidence supporting the potential role of hypoxia modification in selected clinical settings [29]. However, clinical findings have not been uniformly positive. More recently, the NIMRAD randomized Phase III trial provided further clinically relevant evidence regarding hypoxia-targeting radiosensitisation in head and neck cancer. The trial evaluated the addition of nimorazole to radiotherapy in patients with locally advanced head and neck squamous cell carcinoma, including an assessment according to tumor hypoxia status. The addition of nimorazole did not result in a statistically significant improvement in locoregional control or overall survival, including in patients with hypoxia-high tumors. These findings highlight the challenges associated with translating the well-established biological rationale and encouraging preclinical activity of hypoxia-targeting radiosensitisers into consistent clinical benefit. They also reinforce the importance of appropriate patient selection, reliable hypoxia biomarkers, and optimization of treatment and drug-delivery strategies when evaluating hypoxia-targeted radiosensitisation in future clinical studies [25].

Functional imaging techniques, particularly positron emission tomography (PET) using hypoxia-specific tracers such as fluorinated nitroimidazoles, offer a potential solution for identifying hypoxic tumor subregions. These approaches may enable stratification of patients most likely to benefit from hypoxia-targeting radiosensitizers, as well as the implementation of adaptive strategies such as dose painting. Although biomarker-based hypoxia assessment was incorporated in the NIMRAD trial, patient-selection strategies remain heterogeneous across clinical studies. Further refinement and validation of hypoxia biomarkers may therefore be needed to identify the patient populations most likely to benefit from hypoxia-targeting radiosensitisation.

### 4.4. Future Perspectives and Emerging Approaches

Given that most studies included in the present review investigated external beam radiotherapy, the following emerging modalities and technological approaches should be considered primarily as future perspectives rather than as evidence-supported clinical applications of hypoxia-targeting radiosensitization. Recent developments in drug delivery systems suggest additional opportunities to improve the effectiveness of hypoxia-targeting radiosensitizers. Nanoparticle-based platforms, PEGylated compounds, and multifunctional theranostic systems have been developed to enhance tumor targeting, improve pharmacokinetics, and increase intracellular delivery. These approaches may be particularly relevant in heterogeneous tumors, where spatial variations in oxygenation and microenvironmental conditions can influence drug distribution and treatment response [30].

Beyond improving pharmacokinetics, advanced delivery systems may help address one of the main limitations of hypoxia-targeting radiosensitizers, namely the difficulty of achieving sufficient drug concentration within poorly perfused hypoxic regions. Nanocarriers can increase tumor accumulation, prolong local retention, and potentially reduce systemic exposure. Controlled-release formulations may further improve the temporal coordination between drug availability and irradiation, which is particularly relevant for radiosensitizers whose activity depends on local oxygenation and treatment timing. Localized delivery approaches, including implantable or patient-specific devices, may provide an additional strategy to increase drug concentration at the tumor site while limiting off-target toxicity. However, these approaches remain largely preclinical and require further evaluation of biodistribution, release kinetics, biocompatibility, and treatment scheduling before clinical implementation.

Recent advances in RT delivery further complicate and, at the same time, expand the potential role of hypoxia-targeting strategies. Hypofractionated regimens and stereotactic body radiotherapy (SBRT), delivering high doses per fraction, may potentially alter the relative contribution of hypoxia to treatment resistance. While higher doses may partially overcome hypoxia, resistant subregions may still persist, particularly in heterogeneous tumors. Similarly, emerging modalities such as FLASH radiotherapy introduce additional complexity, as ultra-high dose rates may transiently deplete oxygen and modify radiochemical interactions. The interplay between these novel RT paradigms and hypoxia-targeting radiosensitizers remains largely unexplored and represents an important area for future research. In parallel, particle therapy, characterized by higher linear energy transfer (LET), may exhibit reduced dependence on oxygen, raising questions about the relative benefit of hypoxia-targeting approaches in these settings.

An emerging strategy to improve the delivery of radiosensitizers involves the use of advanced manufacturing technologies such as three-dimensional (3D) printing. Additive manufacturing enables the fabrication of patient-specific devices with controlled geometry and composition, which can be engineered to locally deliver therapeutic compounds. In radiotherapy, 3D-printed devices may act as carriers for hypoxia-targeting radiosensitizers, allowing spatially controlled drug release directly at the tumor site. This approach is particularly relevant in brachytherapy, where customized applicators or spacers could enhance local radiosensitization. By enabling localized delivery, 3D printing may help reduce off-target effects and improve tumor-specific accumulation. Although still at an early stage, this strategy represents a promising direction for integrating RT with spatially optimized drug delivery [31].

In parallel, the biocompatibility and safety of radiosensitizers and their delivery systems remain critical aspects for clinical translation. Nitroimidazole-based compounds and related formulations require careful evaluation in terms of toxicity, biodistribution, and long-term effects. The use of biocompatible materials and controlled-release systems is essential to ensure safe and effective delivery. In this context, integrating material science with biological and toxicological assessment is key to optimizing therapeutic performance. Achieving a balance between efficacy and safety remains a central requirement for translating these strategies into clinical practice [32].

### 4.5. Limitations and Research Needs

Several limitations should be considered when interpreting these findings. First, the included studies show a high degree of heterogeneity in experimental models, tumor types, endpoints, and treatment protocols. This limits direct comparability across studies. Second, a substantial proportion of the available evidence is derived from preclinical models. These models may not fully capture the complexity of the tumor microenvironment and treatment response observed in clinical settings. The limited number of clinical investigations and the variability in reported endpoints, including survival-related outcomes and imaging-based metrics, further complicate the interpretation of results. In addition, variability in pharmacokinetics, systemic exposure, extravascular transport, tumor uptake and retention, treatment schedule, and hypoxia-dependent drug activity may influence the observed effectiveness of nitroimidazole-based radiosensitizers and other hypoxia-targeting strategies. More broadly, limited clinical translation may reflect the combined effect of pharmacokinetic constraints, heterogeneous drug penetration into hypoxic tumor regions, variability in the degree and spatial distribution of hypoxia, difficulties in optimizing drug–radiotherapy scheduling, and the need for reliable patient-selection strategies. These factors may reduce the consistency of radiosensitizing effects observed in clinical settings despite promising preclinical results. Within the scope of the present review, clinical evidence was limited, and this may partly reflect the restricted search period, eligibility criteria, and overall number of included studies. Therefore, the findings should not be interpreted as a comprehensive assessment of the entire clinical development landscape of hypoxia-targeting radiosensitizers. Furthermore, the included literature was predominantly based on external beam radiotherapy; therefore, considerations regarding brachytherapy, FLASH radiotherapy, particle therapy, extracellular-vesicle-mediated effects, and 3D-printed delivery systems should be interpreted as emerging translational perspectives rather than conclusions directly supported by the included evidence. In this context, the present review aims to contribute to increasing visibility and integration of existing evidence. In parallel, a deeper understanding of the underlying molecular and radiobiological mechanisms is still needed. This would help identify the most effective conditions for combining these agents with modern RT techniques. Finally, translational progress may also be constrained by limited pharmaceutical development pathways. The establishment of stronger collaborations between academic research and industry stakeholders could improve drug availability, standardization, and clinical implementation.

Future research may benefit from the development of more standardized experimental designs and reporting methodologies to improve comparability across studies. The integration of hypoxia imaging, targeted radiosensitizers, and advanced radiotherapy techniques represents a potential direction for further investigation. In addition, strategies aimed at improving drug delivery, including nanotechnology-based systems and electroporation-assisted approaches, may help to enhance the local effectiveness of radiosensitizers in hypoxic tumor regions. Further clinical studies are warranted to better define the role of these compounds in different tumor types and treatment settings, as well as to identify patient populations that may derive the greatest benefit from hypoxia-targeting strategies.

Overall, the findings of this analysis support the relevance of hypoxia-targeting approaches in the context of radiotherapy, while also highlighting the need for continued research to facilitate their translation into clinical practice. These findings should be interpreted with caution given the variability across studies, but they provide a consistent basis for further investigation.

## 5. Conclusions

Nitroimidazole-based radiosensitizers and other hypoxia-targeting strategies show consistent radiosensitizing effects in preclinical models, while clinical evidence remains limited and heterogeneous. Only three clinical studies were included in the present review, all in HNSCC, and their results were not fully consistent. Advances in imaging and drug delivery approaches may further support their integration into radiotherapy workflows. In this context, the convergence of hypoxia-targeting approaches with precision radiotherapy, functional imaging, and advanced delivery systems may enable a more personalized and biologically guided treatment paradigm. Overall, the translational value of the available studies supports continued efforts to bridge preclinical findings and clinical application, although additional standardized and clinically oriented investigations are needed to better define their role in routine practice.

## Figures and Tables

**Table 1:** Summary of the included preclinical studies investigating hypoxia-targeting strategies and nitroimidazole-based compounds in the context of RT.

Study	Model/Population	Tumor Type	Outcome Category	Radiotherapy Type
Kishimoto et al., 2021 [13]	cancer cell lines	pancreatic cancer	tumor growth	external beam RT
Xu et al., 2022 [14]	TNBC cells + xenograft (MDA-MB-231)	breast cancer (MDA-MB-231)	SER	external beam RT
Mittal et al., 2022 [15]	murine tumor model	non-tumor ovary cell line (CHO) and ovary cancer	tumor response	radionuclide RT (Lu-177)
Leavitt et al., 2024 [16]	cancer cell lines	glioblastoma/HNSCC/lung adenocarcinoma	tumor response	external beam RT
Rashed et al., 2023 [17]	cancer cell lines	HNSCC (FaDu)	SER	external beam RT
Liu et al., 2023 [18]	murine tumor model	colorectal cancer (CT26)	tumor growth	external beam RT
Ge et al., 2023 [19]	murine tumor model	Glioblastoma	tumor inhibition	external beam RT
Liew et al., 2023 [7]	cancer cell lines	colorectal cancer (HCT116)/HNSCC (FaDu)	SER	external beam RT
Chvetsov et al., 2024 [20]	computational model	simulation	modeling	simulated RT
Wu et al., 2024 [21]	cancer cell lines	renal carcinoma	tumor growth	external beam RT
Zhao et al., 2025 [22]	cancer cell lines	cervical cancer (HeLa)	SER/survival	external beam RT
Tian et al., 2025 [23]	murine tumor model	breast cancer (4t1)	tumor growth	external beam RT
Yoshino et al., 2025 [24]	xenograft model	glioblastoma	tumor volume	external beam RT

**Table 2:** Summary of the included clinical studies investigating hypoxia-targeting strategies and nitroimidazole-based compounds in the context of RT.

Study	Model/Population	Tumor Type	Outcome Category	Radiotherapy Type
Thomson et al., 2024 [25]	patients	HNSCC	hazard ratio	external beam RT
Eriksen et al., 2025 [26]	patients	HNSCC	hazard ratio	external beam RT
Grégoire et al., 2026 [27]	patients	HNSCC	hazard ratio	external beam RT

HNSCC, head and neck squamous cell carcinoma.

**Table 3:** Quantitative outcomes extracted from selected studies evaluating the effects of hypoxia-targeting compounds in combination with RT.

Study	Condition/Group	Model	Quantitative Endpoint	Value
Rashed et al., 2023 [17]	IAZA + RT (hypoxia)	FaDu	SER	1.41
	FAZA + RT (hypoxia)		SER	1.09
Xu et al., 2022 [14]	OA + olaparib + RT (in vitro)	MDA-MB-231	SER	1.16–1.65
	OA + olaparib + RT (in vivo)	xenograft model	tumor growth	strong inhibition
Liew et al., 2023 [7]	nitroimidazoles + RT (in vitro)	HCT116/HNSCC	SER	1.3–1.4 *
Yoshino et al., 2025 [24]	RT alone (in vivo)	xenograft model	tumor volume	882%
	compound + RT (in vivo)		tumor volume	288%
Zhao et al., 2025 [22]	NO2-Au6Cu2 + RT (hypoxia)	HeLa	SER	1.43
	H-Au6Cu2 + RT (hypoxia)		SER	1.16
	NO2-Au6Cu2 + RT (normoxia)		SER	1.33
	H-Au6Cu2 + RT (normoxia)		SER	1.33
Thomson et al., 2024 [25]	RT ± nimorazole	patients	hazard ratio (locoregional failure)	0.72 (0.36–1.44)
Eriksen et al., 2025 [26]	RT ± nimorazole	patients	hazard ratio (locoregional failure)	1.16 (0.84–1.59)
Grégoire et al., 2026 [27]	RT ± nimorazole	patients	hazard ratio (locoregional failure)	0.51 (0.27–0.95)

* Values estimated from graphical data (clonogenic survival assays) and reported as mean across compounds. HNSCC, head and neck squamous cell carcinoma; RT, radiotherapy; SER, sensitizer enhancement ratio.

## Data Availability

No new data were created or analyzed in this study. Data sharing is not applicable to this article.
